# A Novel Semi-Supervised Method of Electronic Nose for Indoor Pollution Detection Trained by M-S4VMs

**DOI:** 10.3390/s16091462

**Published:** 2016-09-10

**Authors:** Tailai Huang, Pengfei Jia, Peilin He, Shukai Duan, Jia Yan, Lidan Wang

**Affiliations:** College of Electronic and Information Engineering, Southwest University, Chongqing 400715, China; 18580465830@163.com (T.H.); qaz321123@email.swu.edu.cn (P.H.); duansk@swu.edu.cn (S.D.); yanjia119@163.com (J.Y.); ldwang@swu.edu.cn (L.W.)

**Keywords:** electronic nose, semi-supervised learning, multi-classification, S4VMs

## Abstract

Electronic nose (E-nose), as a device intended to detect odors or flavors, has been widely used in many fields. Many labeled samples are needed to gain an ideal E-nose classification model. However, the labeled samples are not easy to obtain and there are some cases where the gas samples in the real world are complex and unlabeled. As a result, it is necessary to make an E-nose that cannot only classify unlabeled samples, but also use these samples to modify its classification model. In this paper, we first introduce a semi-supervised learning algorithm called S4VMs and improve its use within a multi-classification algorithm to classify the samples for an E-nose. Then, we enhance its performance by adding the unlabeled samples that it has classified to modify its model and by using an optimization algorithm called quantum-behaved particle swarm optimization (QPSO) to find the optimal parameters for classification. The results of comparing this with other semi-supervised learning algorithms show that our multi-classification algorithm performs well in the classification system of an E-nose after learning from unlabeled samples.

## 1. Introduction

Pollution attracts more and more attention as people grow more highly aware of air quality issues. As a result, it is important to detect indoor air pollution effectively. Electronic nose (E-nose) is a metal device that includes a gas sensor array and a processing unit carrying artificial intelligence algorithms. It is usually used in gas analysis problems [1,2,3] and has turned out to be effective. According to previous studies, E-nose has been applied in many fields such as environmental monitoring [4,5], food detection [6,7,8], dangerous objects detection [9], disease diagnosis [10,11,12,13], and aerospace applications [14].

As a branch of gas detection, indoor gas pollution has led to many health problems for people that spend a large amount of their time indoors. Furthermore, if a person inhales too much polluted air unconsciously, it will incur health problems in many aspects. On the other hand, indoor pollution gases such as formaldehyde, toluene, and carbon monoxide are hard to detect and classify in normal ways. So it has become a hot topic to find an effective way to detect indoor gas pollution. Our previous research has proven that E-nose performs well in analyses of these indoor pollution gases [15,16]. 

To improve the performance of E-nose, researchers have proposed many strategies. One focus is on finding new material to build more advanced sensor arrays for E-nose, because sensor arrays usually have many limitations when applied to different fields. It is often the case that sensor arrays respond very quickly to one type of gas, however, they lack sensitivity to another. As result, many sensor arrays have been proposed to improve the performance of E-nose, such as electrochemical, metal oxide, conducting polymer, and coustic wave sensor arrays [17]. They each have advantages in specific fields. For example, metal oxide sensors have a very good response to some gases on the order of sub ppm levels, and electrochemical sensors have good performance in robust as well as low consumption settings. Also, electrochemical sensors can operate at room temperature while other types of sensors often need specific operating temperatures. Moreover, there are many new types of sensor arrays such as colorimetric and optical sensors that are also applied to E-nose [18,19], and they can improve the performance of E-nose significantly. On the other hand, improving the effectiveness of data processing can also help to improve the performance of E-nose. The data processing can be roughly divided into two groups: feature extraction and classification algorithm. In the practical analysis of E-nose, the figures for sample data can be numerous. This makes it hard to deal with data and may increase the running time. Thus, feature extraction as a valid method to reduce the dimension of samples has been introduced for the treatment of samples. Many effective feature extraction methods have been applied for E-nose; for example, principal component analysis (PCA) [20,21] uses an orthogonal transformation to convert a set of observations of possibly correlated variables into a set of values of linearly uncorrelated variables, which can significantly reduce the dimensions of target samples. Another way to enhance the efficiency of an E-nose system is to use advanced analysis algorithms. In the past, researchers often used genetic algorithm [22,23,24] as the major algorithm for classification. However, as samples become more and more complex, these algorithms have been replaced due to their longer analysis times and lower efficiencies. By contrast, many new algorithms such as support vector machines (SVMs) [25,26] and artificial neural networks (ANNs) [27,28] have been introduced to this field.

As a mature technique for classification [29,30], SVM performs well in binary (two-class) classification problems. However, when it comes to gas classification, the number of gases to classify is often more than two, which increases the complexity of the classification. Thus, the use of a single SVM is always avoided to solve multi-class problems directly. On the contrary, it is useful to use a combination of several binary SVM classifiers to solve a given multi-class problem. Researchers have proposed many strategies such as winner-takes-all (WTA-SVM), one-versus-one method implemented by max-wins voting (MWV-SVM), directed acyclic graph procedure (DAG-SVM) [31], and error-correcting codes [32].

On the other hand, SVM often needs to set parameters to reach its best performance. Thus, the use of particle swarm optimization (PSO) [33,34] and QPSO [35] as optimization algorithms to find the best parameters have been applied to the classification methods. Our previous work has proven that these optimization methods can obviously improve the classification rate of E-nose [36].

However, in the real world it is not easy for E-nose to classify different gases under the interference of unlabeled samples. This is because the unlabeled samples are more complex than the labeled samples used in the laboratory. However, in the sampling experiments, there are some cases where the label information of the samples is lost due to mistakes of the operators; for example, where label information is not written on the tag. This leads to the waste of experimental samples. On the other hand, there is plenty of information on unlabeled samples, which can effectively enhance the performance of E-nose [37]. Additionally, unlabeled samples are often easier to obtain and require less time to train the E-nose. The performance of unlabeled samples is only slightly worse than that of labeled samples. Thus, the addition of unlabeled samples is an alternative method to enhance the performance of E-nose. 

Researchers have explored many methods in order to make full use of unlabeled samples as well as minimize the risk of error accumulation. These methods can be roughly divided into three classes:

(1) Active learning: This method is able to select the data to enhance its performance from which it learns. Thus it can achieve ideal accuracy with fewer training labels [38,39].
(2) Transfer learning: These techniques can gain knowledge from related but different tasks to achieve better accuracy in the main task [40,41,42]. However, this often needs sufficient labeled data to provide enough accurate knowledge.
(3) Semi-supervised learning (SSL): This learning paradigm focuses on using a small number of labeled data to determine the label of the data with the help of a large number of unlabeled data [43,44].

Based on the actual situation, we chose semi-supervised learning for improving E-nose performance, because it is easy to obtain many unlabeled samples. The rest of this paper is organized as follows: Section 2 introduces the E-nose system, experimental procedure, and the data set of this paper; Section 3 presents the theory of the S4VMs technique and our enhancement algorithm; Section 4 describes the results of multi-classification S4VMs (M-S4VMs) while it is used for training the classification system of E-nose to distinguish target pollution gases, and to compare with other semi-supervised algorithms. Finally, we draw our conclusion of this paper in Section 5.

## 2. E-Nose System and Experiments 

A concrete description of the E-nose system and the experimental procedure has been expounded in our previous research [37]. Here we simply describe the details which are different from the previous experiment.

In this experiment, we selected three common indoor pollution gases to distinguish. They were carbon monoxide (CO), toluene (C_7_H_8_), and formaldehyde (CH_2_O). We applied a spectrophotometric method and gas chromatography (GC) to determine the concentration of these three gases. The real concentration of the three gases are shown in Table 1. 

Another difference in this paper is the data set. To prove the efficiency of using a semi-supervised algorithm in complex multi-gas classification, we set different proportions of labeled and unlabeled data. The total data was equally divided into two groups, which was used for training and testing, respectively. Therefore, there were 501 samples of complex multi-gases in the training set. Then, we changed the proportion of the data used as labeled data, while the unlabeled data was also changed to keep the total data even. In this paper, we tested the algorithm with a wide range of unlabeled data rates ranging from 10% to 90%. Typical examples of these data sets is shown in Table 2, Table 3 and Table 4. 

## 3. M-S4VMs Technique

### 3.1. S4VMs

S4VMs is an enhancement algorithm generated from S3VMs, which is a branch of transductive support vector machines (TVSM). It has been demonstrated by Yufeng Li and Zhihua Zhou [45] that S4VMs have more advantages in using unlabeled samples than S3VMs and TSVM, and has lower risk with the use of unlabeled samples. The principle of S4VMs is as follows:

First, suppose ${\left\{{\hat{y}}_{t}\right\}}_{t}^{T}=1$ as the predictor of multiple low-density separators, the ground-truth label assignment is $y^{*}$ , and let $y^{svm}$ define the predictions of inductive SVM on $y\in {\left\{\pm 1\right\}}^{u}$ as unlabeled data. For each label assignment, $\mathrm{earn}(y,y^{*},y^{svm})$ and $\mathrm{lose}(y,y^{*},y^{svm})$ are the increased and decreased accuracy which are used to compare with the inductive SVM, respectively. The next step is to improve the performance over the inductive SVM through y; this step can be transformed into an optimization problem shown as Equation (1):

(1) $$\max\{\mathrm{earn}(y,y^{*},y^{\mathrm{svm}})-\lambda \mathrm{lose}(y,y^{*},y^{\mathrm{svm}})\},\;y\in {\left\{\pm 1\right\}}^{u}$$

In this equation, λ is a parameter to trade off how much risk it will undertake during the process. In order to simplify the calculation, we set $\mathrm{earn}(y,y^{*},y^{\mathrm{svm}})-\lambda \mathrm{lose}(y,y^{*},y^{\mathrm{svm}})$ as $J(y,\hat{y},y^{svm})$ .

To solve Equation (1), we must know y, $y^{*}$ , and $y^{svm}$ . According to the parameter set, y is the labeled samples we already know, and $y^{svm}$ is the prediction of unlabeled samples. But the ground-truth $y^{*}$ is unknown, which makes it difficult to complete Equation (1). Thus, we assume that the ground-truth boundary $y^{*}$ can be gained by a low-density separator in ${{\{\hat{y}}_{t}\}}_{t=1}^{T},\;\mathrm{i.e.},\;y^{*}\in M={{\{\hat{y}}_{t}\}}_{t=1}^{T}$ . We set $\bar{y}$ to optimize the worst-case improvement over the inductive SVM, and then $\bar{y}$ can be deduced in Equation (2):

(2) $$\bar{y}={\arg\;\max\;\min\;J(y,\hat{y},y}^{\mathrm{svm}}),y\in {\left\{\pm 1\right\}}^{u},\hat{y}\in M$$

Here is a theorem which shows that the hypothesis is correct. This theorem is shown below:

**Theorem 1.**  *If* $y^{*}\in {\left\{{\hat{y}}_{t}\right\}}_{t=1}^{T}$ *and* λ≥1 *, the accuracy of* $\bar{y}$ *is never worse than that of* $y^{svm}$ .

Via Theorem 1 we can get Proposition 1: If $y^{*}\in {\left\{{\hat{y}}_{t}\right\}}_{t=1}^{T}$ and λ≥1 , the accuracy of y is never worse than that of $y^{svm}$ , as long as the accuracy of y satisfies $\min_{\hat{y}\in M}J(y,\hat{y},y^{svm})\geq 0$ .

On the other hand, $\mathrm{earn}\;(y,y^{*},y^{svm})$ and $\mathrm{lose}(y,y^{*},y^{svm})$ can be expressed as Equations (3) and (4) because they are linear functions of y :

(3) $$\mathrm{earn}(y,y^{*},y^{svm})=\sum_{j=1}^{u}I(y_{j}=y_{j}^{*})I(y_{j}^{*}\neq y_{j}^{svm})=\sum_{j=1}^{u}\frac{1+y_{j}y_{j}^{*}}{2}\frac{1-y_{j}^{svm}y_{j}^{*}}{2}$$

(4) $$\mathrm{lose}(y,y^{*},y^{svm})=\sum_{j=1}^{u}I(y_{j}\neq y_{j}^{*})I(y_{j}^{*}=y_{j}^{svm})=\sum_{j=1}^{u}\frac{1-y_{j}y_{j}^{*}}{2}\frac{1+y_{j}^{svm}y_{j}^{*}}{2}$$

In order not to lose generality, let $J(y,\hat{y},y^{svm})=c_{t}^{\prime }y+d_{t}$ . Then Equation (2) can be expressed as:

(5) $$\max\;\theta \;s.t.\;\theta \leq c_{t}^{\prime }y+d_{t},\forall t=1\cdots T,\theta ,y\in {\left\{\pm 1\right\}}^{u}$$

Although Equation (5) is an integer linear programming, there is no need to obtain the optimal solution to achieve our target based on Proposition 1, therefore a simple heuristic technique is introduced to solve Equation (5). In particular, we relax the integer constraint of y in Equation (5) to [−1,1]^u^ and project back to the integer solution with minimum distance to solve this convex linear programming. Then the output will be replaced when $y^{svm}$ is larger than the result of the integer solution. The final solution clearly satisfies Proposition 1. 

It is not difficult to incorporate prior knowledge on low-density separators into this framework. In order to constrain Equation (5), we employ a dual variable α to complete this target. According to the karush–kuhn–tucker (KKT) condition, we can replace Equation (5) by Equation (6), shown below:

(6) $$\max\min\sum_{t=1}^{T}\alpha_{t}(c_{t}^{\prime }y+d_{t})$$

where $\alpha_{t}$ is proposed as a probability and ${\hat{y}}_{t}$ is in accordance with the ground-truth solution. Thus, while the probabilities α are available for prior knowledge, it can learn the optimal y to improve its performance to the target in Equation (6), by means of known α .

Then set $h(f,\hat{y})$ to represent the function to be minimized by the objective function of S3VMs:

(7) $$h(f,\hat{y})=\frac{{\left\|f\right\|}_{h}}{2}+C_{1}\sum_{i=1}^{l}l(y_{i},f(x_{i}))+C_{2}\sum_{j=1}^{u}l({\hat{y}}_{j},f({\hat{x}}_{j}))$$

In order to gain multiple large-margin low density separators ${{\{f}_{t}\}}_{t=1}^{T}$ and the corresponding label assignments ${{\{\hat{y}}_{t}\}}_{t=1}^{T}$ , we construct Equation (8) to minimize Equation (7):

(8) $$\min\sum_{t=1}^{T}h(f_{t},{\hat{y}}_{t})+M\Omega ({\left\{{\hat{y}}_{t}\right\}}_{t=1}^{T}),\{f_{t},{\hat{y}}_{t}\in \beta \}$$

where *T* represents the number of separators, M represents a large constant enforcing large diversity, and Ω is a quantity of penalty about the diversity of the separators. It is not hard to find that minimizing Equation (7) favors not only the separators with large-margins but also large diversity.

Then, we consider Ω ${{\{\hat{y}}_{t}\}}_{t=1}^{T}$ as the sum of pairwise terms, which can be expressed as Equation (9):

(9) $$\Omega \;({{\{\hat{y}}_{t}\}}_{t=1}^{T})=\sum_{1\leq t\neq \tilde{t}\leq T}I(\frac{{\hat{y}}_{t}^{\prime }{\hat{y}}_{\tilde{t}}}{u}\geq 1-\varepsilon )$$

In this equation, *I* is the identity function and ε∈[0, 1] is a constant when the other penalty quantities are also applicable.

In order to let the outcome be more acceptable, suppose that *f* is a linear model, which can be expressed as $f(x)=w^{\prime }\phi (x)+b$ , where ϕ(x) is induced by the kernel k, which serves as feature mapping. Hence, Equation (10) can be deduced as follows:

(10) $$\begin{array}{l} \min\sum_{t=1}^{T}\left(\frac{1}{2}{\left\|w_{t}\right\|}^{2}+C_{1}\sum_{i=1}^{l}\varepsilon_{i}+C_{2}\sum_{j=1}^{u}{\hat{\varepsilon }}_{j}\right)+M\sum_{1\leq t\neq \tilde{t}\leq T}I(\frac{{\hat{y}}_{t}^{\prime }{\hat{y}}_{\tilde{t}}}{u}\geq 1-\varepsilon );\\ {\left\{w_{t},b_{t},{\hat{y}}_{t}\in \beta \right\}}_{t=1}^{T}\\ y_{i}(w_{t}^{\prime }\phi (x)+b_{t})\geq 1-\varepsilon_{i},\varepsilon_{i}\geq 0;{\hat{y}}_{t,j}(w_{t}^{\prime }\phi ({\hat{x}}_{j})+b_{t})\geq 1-\varepsilon_{j},\varepsilon_{j}\geq 0;\\ \forall i=1,...,\forall j=1,...,u,\forall t=1,...T, \end{array}$$

In which ${\hat{y}}_{t,j}$ is the jth parameters of ${\hat{y}}_{t}$ . If Equation (10) is non-convex then implementation of this method will be presented as follows.

Simulated annealing (SA) [46,47] is an effective method to gain global solutions from objective functions through multiple local minima, which has been proven by Kirkpatrick and V. Černý. Additionally, SA has advantages in replacing current solutions by random nearby solutions according to the value difference between global parameters and function targets in its step. If a global parameter is large, random change will take place in the current solution. On the contrary, if the global parameter is going towards zero, the changes of the current solution will also gradually decline. Laarhoven and Aarts [48] have demonstrated that the convergence analysis of the global solution approaches one when the SA process is extended.

According to Sindhwani [49], a deterministic local search method is used for reducing the low convergence rate of the original SA. Particularly, once ${{\{\hat{y}}_{t}\}}_{t}^{T}=1$ is fixed, multiple individual SVM subroutines will solve ${{\{w}_{t}{,b}_{t}\}}_{t=1}^{T}$ ; and when ${{\{w}_{t}{,b}_{t}\}}_{t=1}^{T}$ is fixed, ${{\{\hat{y}}_{t}\}}_{t}^{T}=1$ is updated according to the local binary search, repeating until convergence.

### 3.2. Multi-Classifier Strategy

However, this type of S4VMs is only a binary (two-class) classification. When it is used for gas sample classification, it is always necessary to construct multi-classifiers. 

According to Kai-Bo Duan and S. Sathiya Keerthi [50], two popular methods for doing this are as follows:

(1) Winner-takes-all strategy SVM (WTA-SVM): this method needs *M* binary classifiers. Suppose the function $\rho_{i}$ is the output of the i-th classifier trained by the examples which comes from $w_{i}$ . $\rho_{i}$ divides the samples into two groups, in which the single type examples are the positive class and all other types are the negative class. Then, the positive class will be removed as one class, and the remaining part will repeat the classification and remove steps until all samples have been classified into their group, which often needs *M* times to classify as well as *M* binary classifiers.
(2) Max-wins voting strategy SVM (MWV-SVM): This method requires constructing $\frac{M(M-1)}{2}$ binary classifiers in which each binary classifier corresponds to every pair of distinct classes. Assuming that a binary classifier is $c_{ij}$ , it is trained by the negative and positive samples taken from sample data $w_{i}$ . When there is a new sample x, $c_{ij}$ will determine if this sample belongs to class $w_{i}$ , and the vote for class $w_{i}$ will add or decrease by one according to the result of $c_{ij}$ . After each of the $\frac{M(M-1)}{2}$ binary classifiers finishes its voting, MWV will allocate x to the class based on the side having the largest number of votes.

### 3.3. M-S4VMs Technique

Based on the original S4VMs, we first improve its performance by constructing a multi-classifier to classify three different gases. Although S4VMs can solve problems with unlabeled data, we want this algorithm to not only use labeled data to train its models for classification, but also add unlabeled data into its learning process. Thus we propose a novel method to improve the performance of S4VMs to complete this target. First, we train S4VMs using labeled data as usual, and classify the unlabeled data through this model. Secondly, S4VMs will add labels to these unlabeled data and calculate the error rate. Then, we add unlabeled data into the labeled data with its label one by one. Finally, we retrain S4VMs with new labeled data to gain a new model to classify the test data.

To gain better performance of S4VMs, we also used an optimization method called QPSO to find the best fit parameters for M-S4VMs. It has been demonstrated that QPSO has fairly good performance in global optimization.

We called our enhanced S4VMs as M-S4VMs, and the steps of the M-S4VMs algorithm (Algorithm 1) is shown as follows:
**Algorithm 1 (M-S4VMs algorithm):****Step 1:** Randomly generate initialized parameters of M-S4VMs;**Step 2:** Train the M-S4VMs through labeled data, then use this model to classify unlabeled samples, finally add this unlabeled data to the labeled data with their label. Retrain the M-S4VMs and get the error rate by classifying the test date. Feedback error rate to QPSO;**Step 3:** Calculate the best fit parameters from the error rate, and update the particles to gain modified parameters;**Step 4:** Return new parameters to M-S4VMs;**Step 5:** Loop Step 2 to Step 4 until the error rate meets the threshold or the loop time arrives to a preset number;**Step 6:** Output the best fitness and classify accuracy rate.



## 4. Results and Discussion

**Figure 1:**
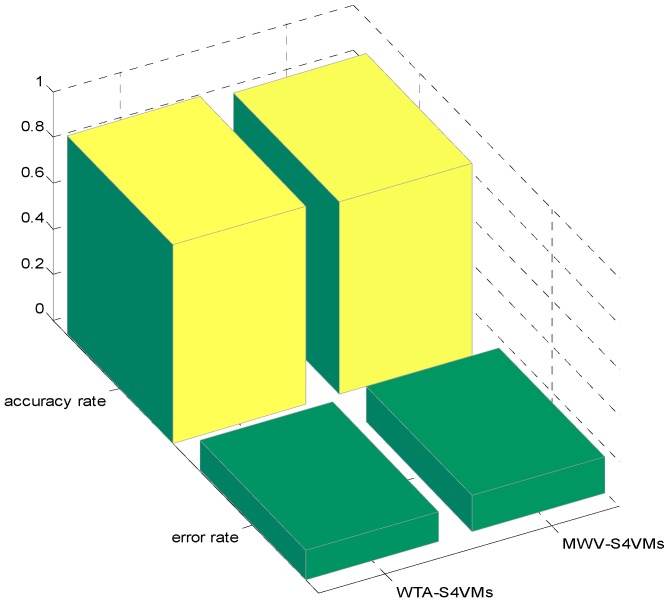
Total accuracy of the two multi-classification methods.

**Figure 2:**
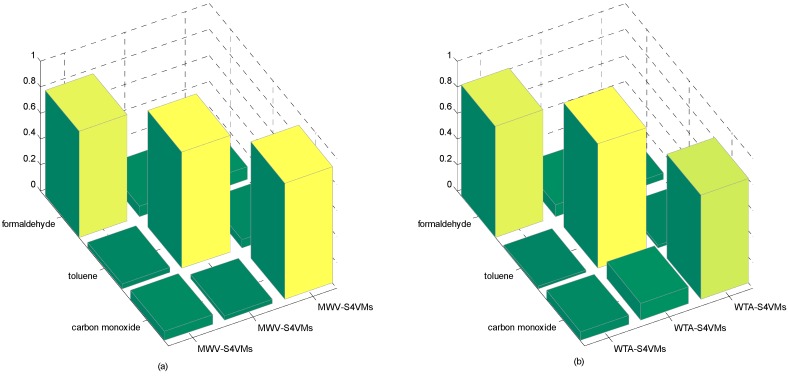
Accuracy of the two multi-classification methods. (**a**) WTA-S4VMs; (**b**) WTA-S4VMs.

In this section, the first step is to decide which multi-class strategy is applied. We designed two different S4VMs with WTA and MWV to test which is better for the current situation. Each test runs ten times to reduce accidental error. Table 5 and Table 6 show the outcomes of these two methods and the classification rate of these two methods for formaldehyde, toluene, and carbon monoxide. Figure 1 below shows the accuracy rate and error rate of WTA-S4VMs and MWV-S4VMs. Figure 2 represents the classification rate of the three gas and the label which has been wrongly labeled.

**Figure 3:**
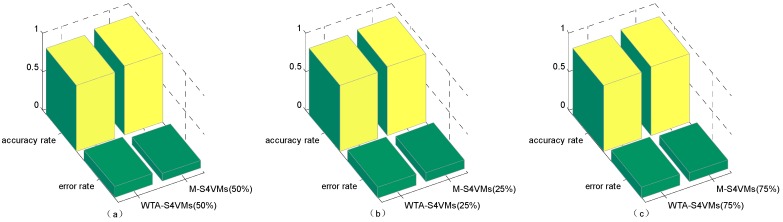
Total accuracy rate of the two algorithms with different unlabeled rates. It illustrates the total classification results for these three target gases by M-S4VMs and WTA-S4VMs when the unlabeled data account for 50% (**a**), 25% (**b**) and 75% (**c**), respectively. And it obvious that no matter what unlabeled rate it is, M-S4VMs always performs better than WTA-S4VMs.

**Figure 4:**
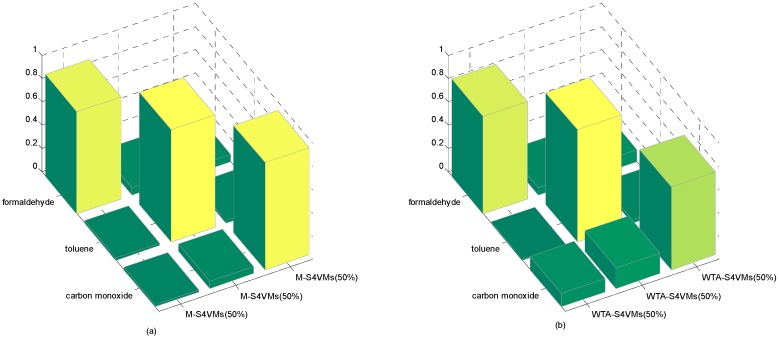
50% unlabeled rate of the two methods. It illustrates the classification results based on three target gases by M-S4VMs (**a**) and WTA-S4VMs (**b**) when the unlabeled data account for 50%. It is obvious that M-S4VMs performs better than WTA-S4VMs especially in the classification of carbon monoxide.

**Figure 5:**
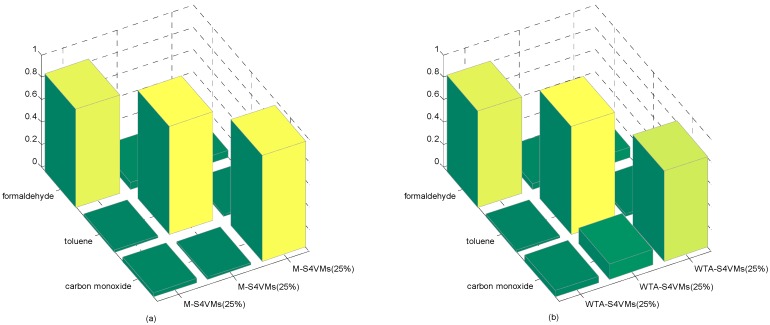
25% unlabeled rate of the two methods. It illustrates the classification results based on three target gases by M-S4VMs (**a**) and WTA-S4VMs (**b**) when the unlabeled data account for 25%. According to the figure, it is clear that M-S4VMs gets better accuracy rate than WTA-S4VMs in 25% unlabeled rate.

**Figure 6:**
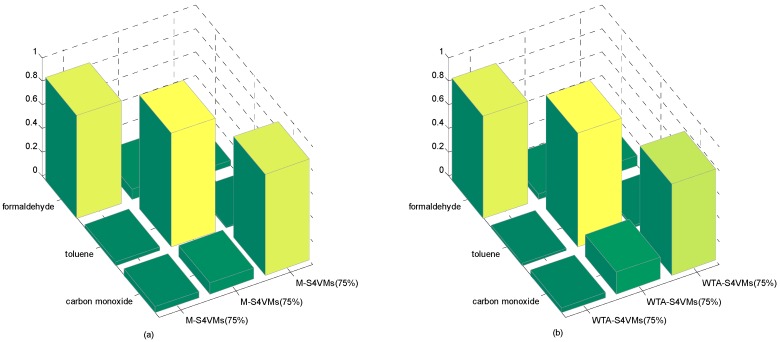
75% unlabeled rate of the two methods. It illustrates the classification results based on three target gases by M-S4VMs (**a**) and WTA-S4VMs (**b**) when the unlabeled data account for 75%. It is clear that M-S4VMs has specific improvement in the classification of carbon monoxide compared with WTA-S4VMs in 75% unlabeled rate.

**Figure 7:**
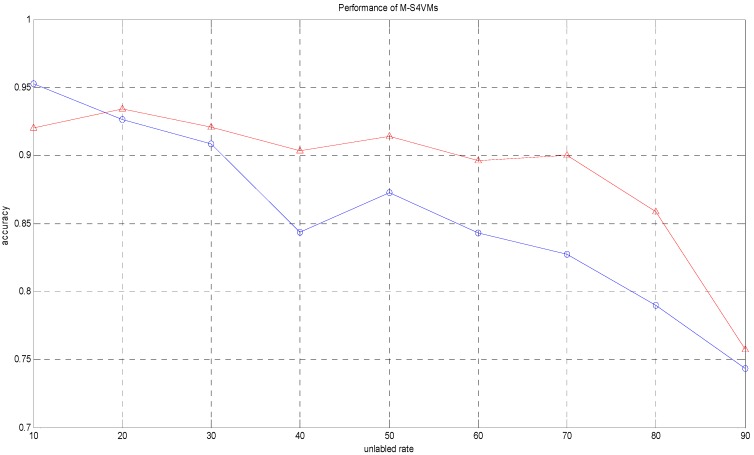
Performance of M-S4VMs with different unlabeled rates. Note: Data1 (blue) represents the accuracy of the first classification without unlabeled sample, and data2 (red) represents the accuracy of M-S4VMs with unlabeled sample in the second classification.

It is clear that WTA-S4VMs has better performance than MWV-S4VMs, so WTA-S4VMs was selected as the major multi-classifier method of M-S4VMs. Then we applied our optimizing methods to WTA-S4VMs. Table 7 illustrates the performance of these two methods and the different classification rates of these two methods. Then Table 8 show the classification rate with different unlabeled sample rates. Figure 3, Figure 4, Figure 5 and Figure 6 show the classification rates of WTA-S4VMs and M-S4VMs with different unlabeled rates as well as the classification rates of the three gases with wrong labeled sample rates. Table 9 and Figure 7 show that the performance of M-S4VMs with different unlabeled rates from 10% to 90% where the total number of labeled samples and unlabeled samples are constant, but the number of unlabeled samples and labeled samples are dynamic as the unlabeled rate changes.

It is easy to see that with unlabeled samples added into the training samples, the performance of S4VMs gains obvious improvement. Additionally, the classification rate of target gases is also improved. This is because the unlabeled data also contains a lot of useful information about classifying different gases. When it is added into the training samples, S4VMs has more samples to modify its prediction model for classification. However, the hidden information in unlabeled samples is limited. In Table 7 it is clear that the improved accuracy from adding unlabeled samples declines as the unlabeled rate increases from 50% to 75%.

In Table 9 and Figure 7, it is obvious that the performance of M-S4VMs decreases quickly at the first classification, but then increases and maintains an ideal level in the second classification. This is because the labeled samples are sufficient at first, so the performance of M-S4VMs in the first classification is quite good. However, as the labeled samples decrease, the performance of M-S4VMs also declines. This shows that with insufficient labeled samples, the M-S4VMs performance cannot achieve its full potential. On the other hand, the unlabeled samples increase at the same time, and M-S4VMs can add unlabeled samples that it has classified into the training samples to modify its model, which obviously improves the performance of M-S4VMs in the second classification. However, when the unlabeled rate is too large, the error accumulation of semi-supervised algorithms will damage the performance of M-S4VMs, which can be observed in the 80% as well as 90% unlabeled rate. In contrast, without unlabeled samples added into the training set, the accuracy of the classification continues to decline.

**Figure 8:**
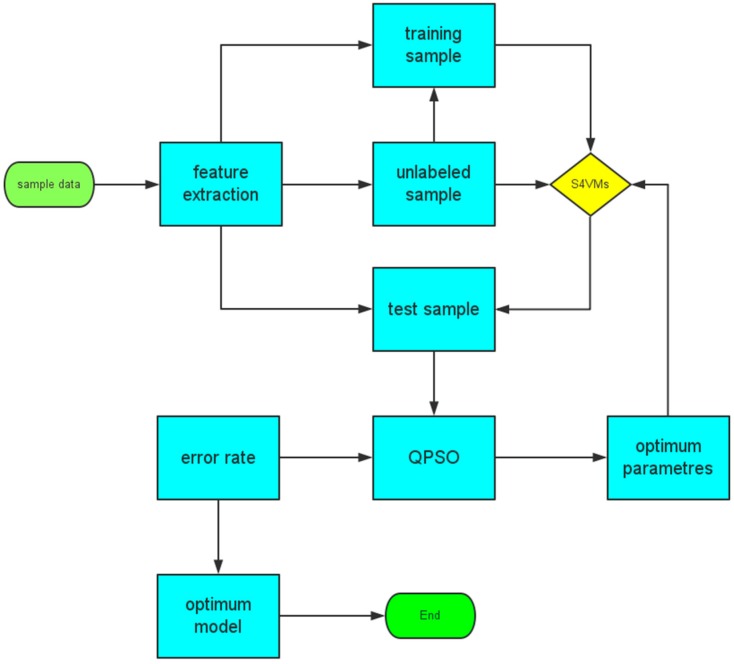
Flow chart of the algorithm.

When M-S4VMs is applied to the E-nose system, there are two optimization parameters (the penalty coefficient and the radius of the kernel function in SVM) that need to be decided. QPSO [51] is a fairly global optimization algorithm which has been proven to be effective in searching for the best parameters for classifiers. Because the M-S4VMs has two parameters, we set the dimension at two and the swarm size as ten. The flow chart of the algorithm is shown in Figure 8 below.

**Figure 9:**
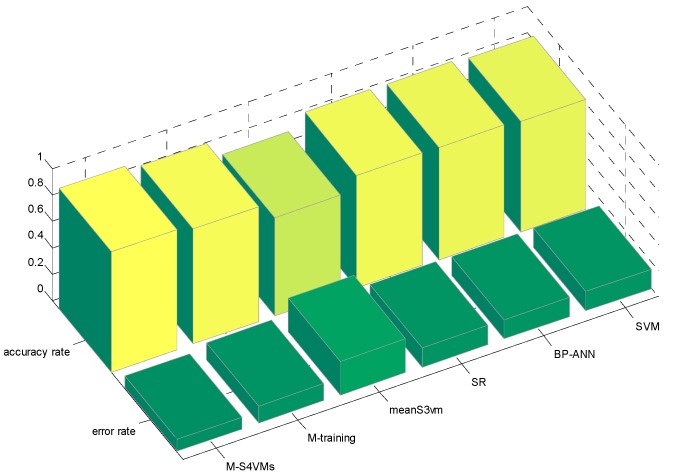
Total accuracy rate of the four semi-supervised algorithms.

**Figure 10:**
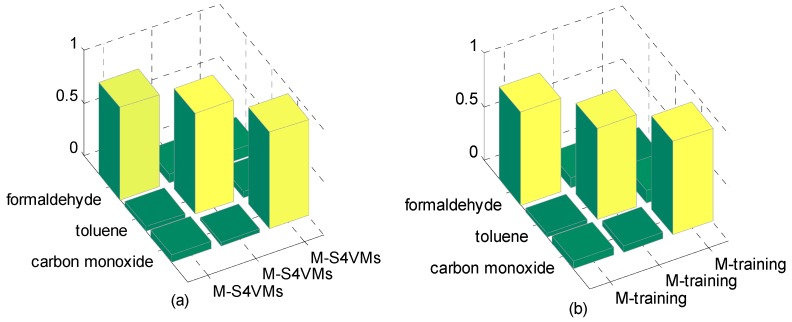

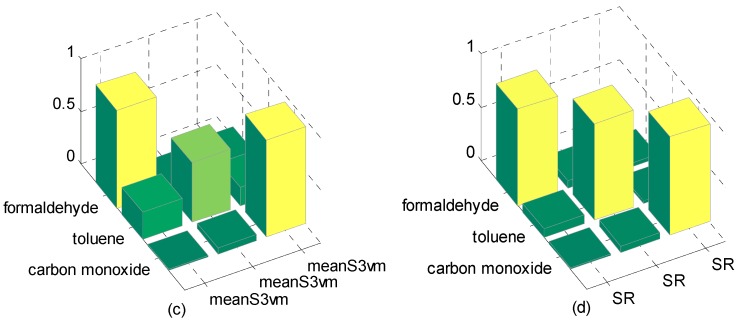
Accuracy rate of the four semi-supervised algorithms. These are the classification results based on three target gases with two normal semi-supervised algorithms which are M-S4VMs (**a**); M-training (**b**); meanS3vm (**c**); SR (**d**), respectively. It is clear that M-S4VMs has the ideal performance in classification. Although other algorithms also performs well in some specific gases classification, M-S4VMs still works better than them in terms of total three gases classification.

**Figure 11:**
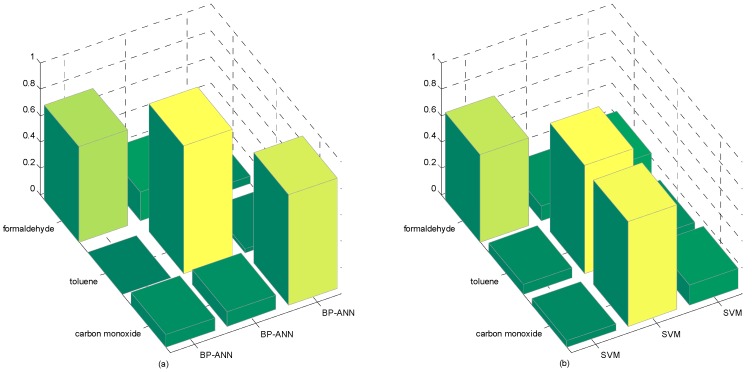
Accuracy rate of the two conventional nonlinear supervised methods. These are the classification results based on three target gases with conventional nonlinear supervised methods which are BP-ANN (**a**) and SVM (**b**). It is obvious that these two algorithms all perform well in specific gas classification. However, their performance get worse while applied to other gases classification.

As a novel classification algorithm, it is necessary to compare with other semi-supervised algorithms; thus, we selected meanS3vm [52], M-training [53,54], and SR [55,56] for comparison. We also added two conventional nonlinear supervised methods, BP-ANN and SVM, into the comparison. Table 10 and Table 11 below show the outcome of the six semi-supervised algorithms and their classification rates of the target gases. Figure 9 and Figure 10 illustrate the accuracy of these six semi-supervised algorithms as well as the classification of target gases and their wrongly labeled sample rates. Figure 11 show the accuracy of the two conventional nonlinear supervised methods.

It is clear that M-S4VMs has a better performance than the other algorithms. For the outcomes of the four different semi-supervised algorithms, M-S4VMs has best performance not only in the minimum but also in the maximum classification rates. When it comes to the average classification rate, M-S4VMs still has the best performance.

We also compared this method with the SR and MeanS3vm methods with respect to running time. The results are shown in Table 12 below.

It is clear that meanS3vm has the longest classification running time, while BP-ANN took the least time to complete its classification. Within these six methods, M-S4VMs also performs very well. Although its running time is slightly longer than SR and BP-ANN, we find that M-S4VMs has a better accuracy rate after classification.

As for the outcomes of the classification rate of target gases, M-S4VMs performs well in classifying toluene as well as carbon monoxide. Conversely, although meanS3vm has the best performance in the classification of formaldehyde and carbon monoxide, it performs poorly in the classification of toluene, where M-S4VMs is significantly better. Summarizing all of the three target gases, M-S4VMs performs better than SR, although a little worse than SR, in classifying formaldehyde. When M-S4VMs was compared with the two conventional nonlinear supervised methods, it was also very obvious that M-S4VMs outweighs SVM and BP-ANN not only in total classification, but also in the classification of each gas except toluene, where BP-ANN only slightly exceeded M-S4VMs. 

## 5. Conclusions 

An E-nose consisting of a sensor array and an artificial algorithm can identify typical patterns to gas samples. In order to detect different gases precisely, it is essential to train the E-nose with enough samples. In general, researchers often use labeled samples to train the E-nose, which can help it gain ideal accuracy. However, this usually requires a large number of labeled samples and a long time to train the E-nose. By contrast, in the real world, unlabeled samples are easier to find and require less time than labeled samples in the training of an E-nose. Thus, it has become a hot topic to introduce unlabeled samples into E-nose training.

In this paper, we focus on making full use of unlabeled samples based on S4VMs, but original S4VMs can only solve binary classification problems. Therefore, we first change S4VMs into a multi-classifier by a popular multi-classifier SVM construction strategy. Then we propose a novel method to add unlabeled samples into training samples to improve the classification ability of S4VMs. These do not only use labeled samples to train the E-nose to classify unlabeled samples, but also use these unlabeled samples to revise its model, which can improve the performance of the E-nose. The results of the experiment with these multi-gases and the comparison with other semi-supervised algorithms have proven that this method can improve the performance of S4VMs and gain classification information from the unlabeled samples. However, the information in unlabeled samples is limited, so the classification rate will decline as the number of unlabeled samples increases. This is also clear for our experimental results; if the unlabeled rate increases too much, the accuracy of the algorithm declines gradually.

In conclusion, it is valid to add unlabeled samples into training samples to enhance the performance of the E-nose, especially when labeled samples are insufficient. Moreover, unlabeled samples can increase the variety of the training samples, which can make the E-nose perform better in real world applications. Additionally, unlabeled samples are easier to obtain and use, which can reduce the cost of the E-nose. However, there are still some problems that remain. For example, reduction of the speed of error accumulation needs further research. Unlabeled samples may be wrongly classified, and when this accumulates, it can lead to the decline of the E-nose’s accuracy as the number of unlabeled samples increases. However, S4VMs has demonstrated that it has a lower classification risk than other semi-supervised methods when it encounters unlabeled samples. All of these results make it obvious that M-S4VMs is an effective semi-supervised method for the E-nose, used to classify carbon monoxide, formaldehyde, and toluene. 

## Figures and Tables

**Table 1:** Concentration of the target gases.

Gases	Concentration Range (ppm)
Carbon monoxide	[4, 12]
Toluene	[0.0668, 0.1425]
Formaldehyde	[0.0565, 1.2856]

**Table 2:** Amount of samples in a data set with 50% unlabeled rate.

Gases	Training Set	Unlabeled Set	Test Set
Carbon monoxide	116	116	116
Toluene	132	132	132
Formaldehyde	253	253	253
All-3	501	501	501

**Table 3:** Amount of samples in a data set with 75% unlabeled rate.

Gases	Training Set	Unlabeled Set	Test Set
Carbon monoxide	58	174	116
Toluene	66	198	132
Formaldehyde	126	380	253
All-3	250	752	501

**Table 4:** Amount of samples in a data set with 25% unlabeled rate.

Gases	Training Set	Unlabeled Set	Test Set
Carbon monoxide	174	58	116
Toluene	198	66	132
Formaldehyde	380	126	253
All-3	752	250	501

**Table 5:** Outcome of WTA-S4VMs and MWV-S4VMs in multi-gases.

	Min	Max	Average
WTA-S4VMs	0.8524	0.8724	0.8692
MWV-S4VMs	0.8276	0.8678	0.8438

Note: the accuracy rate is defined as follows: Accuracy = (acc1 * n1 + acc2 * n2 + acc3 * n3)/(n1 + n2 + n3), where acc1, acc2, acc3 represent the accuracy of formaldehyde, toluene, and carbon monoxide, respectively. And n1, n2, n3 state the sample number of formaldehyde, toluene, and carbon monoxide.

**Table 6:** Outcome of WTA-S4VMs and MWV-S4VMs in multi-gases.

	Formaldehyde	Toluene	Carbon Monoxide
WTA-S4VMs	0.8575	0.9599	0.8049
MWV-S4VMs	0.8176	0.8998	0.8978

Note: the accuracy rate is defined as follows: accuracy = *L/N*; *L* represents the number of labels given by a classifier which meet the true labels; *N* states the number of labels.

**Table 7:** Outcome of the total accuracy rate at different unlabeled rates.

	Min	Max	Average	Improvement	Unlabeled Rate
WTA-S4VMs	0.8424	0.8724	0.8592	5%	50%
M-S4VMs	0.8972	0.9140	0.9002		
WTA-S4VMs	0.8324	0.8679	0.8579	3%	25%
M-S4VMs	0.8772	0.9070	0.8895		
WTA-S4VMs	0.8324	0.8624	0.8592	3%	75%
M-S4VMs	0.8872	0.9143	0.8867		

**Table 8:** Outcome of the target gases accuracy rate at different unlabeled rates.

	Formaldehyde	Toluene	Carbon Monoxide	Unlabeled Rate
WTA-S4VMs	0.8343	0.9588	0.7104	50%
M-S4VMs	0.8742	0.9599	0.9188	
WTA-S4VMs	0.8575	0.9599	0.8049	25%
M-S4VMs	0.8739	0.9639	0.9438	
WTA-S4VMs	0.8636	0.9602	0.7722	75%
M-S4VMs	0.8687	0.9599	0.8537	

**Table 9:** Outcome of M-S4VMs at different unlabeled rates.

Unlabeled Rate	Accuracy1	Accuracy2
10%	0.9527	0.9200
20%	0.9262	0.9343
30%	0.9086	0.9210
40%	0.8438	0.9037
50%	0.8728	0.9140
60%	0.8430	0.8960
70%	0.8271	0.8999
80%	0.7896	0.8589
90%	0.7435	0.7576

Note: accuracy1 represents the accuracy of the first classification without unlabeled samples, and accuracy2 represents the accuracy of M-S4VMs with unlabeled samples.

**Table 10:** Outcomes of the six semi-supervised algorithms.

	Min	Max	Average
M-S4VMs	0.8967	0.9166	0.9102
M-training	0.8633	0.8755	0.8702
meanS3vm	0.7354	0.7632	0.7448
SR	0.8437	0.8637	0.8535
BP-ANN	0.8425	0.8764	0.8525
SVM	0.8430	0.8258	0.8335

**Table 11:** Outcomes of the classification rates of target gases.

	Formaldehyde	Toluene	Carbon Monoxide
M-S4VMs	0.8742	0.9599	0.9188
M-training	0.8687	0.8555	0.8733
meanS3vm	0.9317	0.5682	0.9277
SR	0.8998	0.8988	0.9188
BP-ANN	0.7222	0.9697	0.8412
SVM	0.6652	0.8223	0.7932

**Table 12:** Running time of classification rate of target gases per second.

	Running Time (s)
M-S4VMs	35.410543
M-training	48.008512
meanS3vm	179.690567
SR	21.677869
BP-ANN	18.648126
SVM	42.281541
